# NIA DIVISION OF BEHAVIORAL AND SOCIAL RESEARCH

**DOI:** 10.1093/geroni/igae098.0334

**Published:** 2024-12-31

**Authors:** Lisbeth Nielsen

**Affiliations:** National Institute on Aging, Baltimore, Maryland, United States

## Abstract

Dr. Nielsen will discuss research priorities of the NIA Division of Behavioral and Social Research. Dr. Nielsen will also be available for small group discussion.

